# Effects of Prenatal Exposure to Coal-Burning Pollutants on Children’s Development in China

**DOI:** 10.1289/ehp.10471

**Published:** 2008-01-30

**Authors:** Deliang Tang, Tin-yu Li, Jason J. Liu, Zhi-jun Zhou, Tao Yuan, Yu-hui Chen, Virginia A. Rauh, Jiang Xie, Frederica Perera

**Affiliations:** 1 Department of Environmental Health Sciences; 2 Columbia Center for Children’s Environmental Health, Columbia University, New York, New York, USA; 3 Chongqing Children Hospital, Chongqing University of Medical Sciences, Chongqing, China; 4 School of Public Health, Fudan University, Shanghai, China; 5 School of Environmental Science and Engineering, Shanghai Jiaotong University, Shanghai, China

**Keywords:** China, coal burning, lead, mercury, neurodevelopment, PAH–DNA adducts, prenatal

## Abstract

**Background:**

Environmental pollutants such as polycyclic aromatic hydrocarbons (PAHs), lead, and mercury are released by combustion of coal and other fossil fuels.

**Objectives:**

In the present study we evaluated the association between prenatal exposure to these pollutants and child development measured by the Gesell Developmental Schedules at 2 years of age.

**Methods:**

The study was conducted in Tongliang, Chongqing, China, where a seasonally operated coal-fired power plant was the major source of ambient PAHs and also contributed lead and mercury to the air. In a cohort of nonsmoking women and their newborns enrolled between March 2002 and June 2002, we measured levels of PAH–DNA adducts, lead, and mercury in umbilical cord blood. PAH–DNA adducts (specifically benzo[*a*]pyrene adducts) provided a biologically relevant measure of PAH exposure. We also obtained developmental quotients (DQs) in motor, adaptive, language, and social areas.

**Results:**

Decrements in one or more DQs were significantly associated with cord blood levels of PAH–DNA adducts and lead, but not mercury. Increased adduct levels were associated with decreased motor area DQ (*p* = 0.043), language area DQ (*p* = 0.059), and average DQ (*p* = 0.047) after adjusting for cord lead level, environmental tobacco smoke, sex, gestational age, and maternal education. In the same model, high cord blood lead level was significantly associated with decreased social area DQ (*p* = 0.009) and average DQ (*p* = 0.038).

**Conclusion:**

The findings indicate that exposure to pollutants from the power plant adversely affected the development of children living in Tongliang; these findings have implications for environmental health policy.

Molecular and epidemiologic research has shown that fetuses and infants are more susceptible than adults to the harmful effects of a variety of environmental contaminants, including polycyclic aromatic hydrocarbons (PAHs) (Perera et al. 2005), mercury [Agency for Toxic Substances and Disease Registry (ATSDR) 1999; Chang 1977; Clarkson 2002; Goldman and Shannon 2001; Harada 1977; Koos and Longo 1976; Marsh 1987; Marsh et al. 1987; National Research Council 2000; Ratcliffe et al. 1996; Satoh 2000], lead (ATSDR 2005; Cory-Schlecta and Schaumburg 2000; Dietrich et al. 1991; Emory et al. 2003; Leggett 1993; Wasserman et al. 1997, 2000), and environmental tobacco smoke (ETS), pesticides, and polychlorinated biphenyls [National Research Council 1993; Neri et al. 2006; Whyatt and Perera 1995; World Health Organization (WHO) 1986]. There is growing evidence that prenatal exposures to air pollutants from combustion of coal and other fossil fuels have adverse effects on fetal growth and early child neurodevelopment. PAHs are toxic, mutagenic, and carcinogenic air pollutants that are generated by the incomplete combustion of fossil fuels such as coal, diesel, and gasoline (Bostrom et al. 2002). PAHs are present in tobacco smoke and grilled or broiled foods, as well as in ambient air. Some PAHs are transplacental carcinogens (Bulay and Wattenberg 1971; Rice and Ward 1982; Vesselinovitch et al. 1975). Adduct concentrations in both maternal and cord blood have been shown to increase with a rise in estimated ambient exposure to PAHs, although there is substantial interindividual variation in adduct formation (Perera et al. 2005; Whyatt et al. 1998). Because PAH–DNA adducts reflect individual variations in exposure, absorption, metabolic activation, and DNA repair, they provide an informative biologic dosimeter that has been associated with risk of cancer (Bartsch et al. 1983; Pelkonen et al. 1980; Tang et al. 2001) and reproductive/developmental effects (Perera et al. 1998, 2007; Tang et al. 2006).

Experimental animal studies have demonstrated that benzo[*a*]pyrene (BaP), a representative PAH, is a reproductive toxicant (Archibong et al. 2002) and produces a variety of neurodevelopmental effects as a result of nervous system damage, including decreased motor activity; neuromuscular, physiologic, and autonomic abnormalities, and decreased responsiveness to sensory stimuli (Saunders et al. 2002, 2003; Wormley et al. 2004b). In epidemiologic studies, prenatal exposure to PAHs has been shown to be associated with reduced birth weight, length, and head circumference (Choi et al. 2006; Perera et al. 1998, 2003; Šrám et al. 2005; Tang et al. 2006). In the present cohort, reduction of head circumference was associated with PAH–DNA adducts in cord blood (Tang et al. 2006). Reduction of weight or head circumference at birth has been correlated with lower IQ as well as poorer cognitive functioning and school performance in childhood (Chaikind and Corman 1991; Chasnoff et al. 1992; Desch et al. 1990; Hack et al. 1991; Lucas et al. 1992; Matte et al. 2001). Several studies have associated PAH exposure with decrements in cognitive development. For example, children born during the years of maximal air pollution in the Czech Republic had learning disorders that were attributed in part to elevated levels of PAHs in the atmosphere from the mining and combustion of coal (Otto et al. 1997). Children in New York City who were more highly exposed to PAHs *in utero* had significantly decreased Bayley Mental Development Index scores and were more likely to be developmentally delayed at 3 years of age (Perera et al. 2006).

Lead and mercury have been shown in multiple studies to be developmental neurotoxins. Lead is produced by the combustion of coal and has been widely used as an additive in gasoline and in paint. Numerous studies have reported an association between lead exposure and decreased IQ scores on various intelligence scales even at low levels of exposure (ATSDR 2005; Canfield et al. 2003; Lanphear et al. 2005; Surkan et al. 2007). Lead has also been associated with antisocial and delinquent behavior (Needleman et al. 1996). Several studies have determined that prenatal exposure to mercury is related to adverse neurodevelopmental outcomes (ATSDR 1999; Chang 1977; Clarkson 2002; Goldman and Shannon 2001; Grandjean et al. 1997; Koos and Longo 1976; National Research Council 2000; Ratcliffe et al. 1996; Satoh 2000).

The present cohort study was conducted in Tongliang, Chongqing, which has a population of around 100,000 and is situated in a basin approximately 3 km in diameter. Before its permanent shutdown in December 2004, a coal-fired power plant located south of the town center operated every year from 1 December to 31 May to compensate for the insufficient hydraulic power during the dry season (Chow et al. 2006; Tang et al. 2006). The plant was not equipped with modern pollution reduction technology and combusted about 25,000 tons of coal during each 6-month period of operation. This report concerns the cohort of children whose gestational period included the months of power plant operation from 1 December 2001 to 31 May 2002.

The main exposure of interest (PAHs) resulted primarily from the burning of coal fuel by the local power plant. In 1995 nearly all domestic heating and cooking units were converted to natural gas, and in 2002 motor vehicles were limited in number. Air monitoring analyses carried out as part of the study showed that PAHs of medium molecular weight (168–266 Da) increased by 1.5–3.5 times during the Tongliang power plant’s operational period (Chow et al. 2006). The plant also emitted lead and mercury. Emissions from coal combustion in China are a major source of lead contamination in China (Guo et al. 1994, 2002; Zajusz-Zubek and Konieczynski 2003; Zhang et al. 2003). The average lead content of Chinese coal is about 1,000 times higher than in U.S. coal (Kunli et al. 2005; Mastalerz and Drobniak 2005). The Chinese government banned lead in gasoline in the year 2000; however, some resuspension of lead-containing dust from the roadside soil may have contributed to lead levels in cord blood. Coal combustion is responsible for an estimated 46% of Chongqing’s total mercury emissions (Wang et al. 2006). The major route of mercury exposure is via ingestion of fish and shellfish contaminated by methylmercury derived from deposition of mercury emitted to the air.

The present study evaluated the association between levels of PAH–DNA adducts, lead, and mercury and 2-year cognitive development as measured by the Gesell Developmental Schedules (GDS) (Gessel and Amatruda 1941). We tested the hypothesis that after adjusting for potential confounders, prenatal exposure to PAHs, lead, or mercury would be associated with lower DQs in motor, adaptive, language, and social areas at 2 years of age.

## Materials and Methods

### Study subjects

Subjects were children born to nonsmoking Chinese women who gave birth at any one of three Tongliang county hospitals between 4 March 2002 and 19 June 2002. The women were selected using a screening questionnaire when they checked in for delivery. Eligibility criteria included non-smoking status, age ≥ 20 years, and residence within 2.5 km of the Tongliang power plant. Of 150 eligible consenting women, 149 completed the interview and contributed a cord blood sample at the time of delivery. The retention rate for the full cohort was 88.7% at the 2-year follow-up. One hundred thirty-three mother–child pairs remained in the study 2 years after the study had begun. All of the 133 children were tested with the GDS. One hundred ten subjects had complete data on all measures and variables required for the analysis. Table 1 presents the demographic characteristics of the 110 children who are the subjects of the present report. As shown in Table 1, this subset did not differ significantly with respect to demographic characteristics from the group not included in the analysis (e.g., maternal age, education, gestational age, birth weight, birth length, and birth head circumference). All subjects signed the consent form approved by the Columbia University Institutional Review Board and Chongqing University of Medical Sciences.

### Personal interview

A trained interviewer administered a 45-min questionnaire after delivery that included demographic information, lifetime residential history (location of birth and duration of each residency), history of active and passive smoking, occupational exposure during pregnancy (chemical exposure, including coal products from hot asphalt or tar roofing material, agriculture herbicides, and insecticides), medication information, alcohol use during each trimester of pregnancy, consumption of PAH-containing meat (frequency of eating fried, broiled, or barbecued meat), and fish consumption (as a potential source of mercury). ETS exposure was measured by a set of questions about timing, frequency, and the amount of exposure to cigarette, cigar, and pipe smoke in the home. Socioeconomic information related to income and education was also collected. As reported in previous studies, the Chinese are reluctant to reveal their true income (Xu et al. 2006); hence, we considered education level a more reliable indicator of socioeconomic status.

### Biological sample collection and analysis

Umbilical cord blood was collected at delivery. The blood samples for PAH–DNA adducts were collected in heparinized BD Vacutainer tubes (10 mL, green) (Becton Dickinson, Franklin Lakes, NJ, USA). The blood samples for mercury and lead analysis were collected in EDTA Vacutainer tubes (4 mL, lavender). Samples were transported to the field laboratory at the Tongliang County Hospital immediately after collection. The buffy coat, packed red blood cells, and plasma were separated and stored at −70°C.

#### PAH–DNA adducts

We analyzed BaP–DNA adducts in extracted white blood cell DNA using a high-performance liquid chromatography (HPLC)/fluorescence method that detects BaP tetraols (Alexandrov et al. 1992; Rojas et al. 1994), modified as described previously (Perera et al. 2007). Briefly, 100 μg of DNA were used for each analysis. Many precautions were taken to avoid the presence of fluorescent contaminants. DNA samples were dissolved in 0.1 N HCl and hydrolyzed at 90°C for 6 hr. The resulting solution was analyzed in a Shimadzu HPLC system with an RF-10Axl spectrofluorometric detector (Shimadzu, Kyoto, Japan). Samples were injected using the Shimadzu S IL-10A automatic sample injector. We calculated tetraol concentrations by comparing the samples analyzed with an external calibration curve generated from the fluorescence peak of a known amount of authentic benzo[*a*]pyrene diol epoxide (BPDE) tetraol standard each time a set of samples was analyzed. Calibration was carried out with DNA from calf thymus alone (background) and spiked with 2, 4, and 8 pg anti-BPDE tetraol. These standard solutions were then treated in the same way as the tested samples. The correlation coefficient was 0.98, and the mean coefficient of variation for analyses repeated on different days was 12%. The detection threshold of BPDE tetraols [r-7,c-10,t-8,t-9-tetrahydroxy-7,8,9,10-tetrahydrobenzo[*a*]pyrene (BaP tetraol I-1) and r-7,t-9,t-10,t-8-tetrahydroxy-7,8,9,10-tetrahydrobenzo[*a*]pyrene (BaP tetraol I-2)] was 0.25 pg (signal-to-noise ratio > 3) so that, in the present study, with 100 μg DNA, this assay could detect 0.25 adducts per 10^8^ nucleotides. Assays were performed on all samples that were of adequate quantity and quality for analysis. All samples were run coded.

#### Cord lead

Portions of umbilical cord blood were sent to the Laboratory of the Department of Occupational Health, School of Public Health, Fudan University in Shanghai, China. Whole blood samples were analyzed per the standard U.S. Environmental Protection Agency (EPA) method, the PE-800 Zeeman atomic absorption spectrometer with Zeeman background correction system (U.S. EPA 2007). The lead standard (1.0 g/L) was purchased from the Shanghai Institute of Testing Technology. Nitric acid of ultrapure reagent grade was purchased from the Shanghai Reagent Factory. All vessels were soaked in 0.3% nitric acid for 24 hr and then washed with double-distilled water. Preparation of standards and samples was carried out under lead-free conditions. One hundred microliters of the sample was diluted 1:1 with 0.1% HNO_3_. We used a PE-800 Zeeman atomic absorption spectrometer with Zeeman background correction system. The wavelength was 283.3 nm, lamp current was 10 mA, slit width was 0.7 nm, and sample volume was 10 μL. For quality control, we used NYCOMED whole blood (batch 905; 400 ± 24 μg/L; Zurich, Switzerland) certified reference material. The determination limit of this method was 0.09 μg/L. The recovery rate of this method in the laboratory is > 92%; the precision is 1.7–3.8%.

#### Cord mercury

Researchers at the Laboratory of the Department of Occupational Health, School of Public Health, Fudan University, also performed the analysis of mercury using the Automatic Mercury Analyzer AMA-254 (Milestone Inc., Monroe, CT, USA) that can directly test total mercury in the samples without any pretreatment. The method complies with U.S. EPA method 7473. A reference standard, BCR 40 (mercury concentration, 0.35 ± 0.06 μg/g), which was developed by the Commission of the European Community (2008), was used to ensure the validity of the method. As required by the method, the first working curve range set by the instrument was used for calibration. Our calibration curve provided parameters within the acceptable range set by the analyzer.

### Measures of child neurodevelopment and covariates

The GDS was designed to provide a neurologic and intellectual evaluation of the infant or child at the time of testing and for making decisions regarding services needed by the infant (Gesell and Amatruda 1941). Unlike tools that assess IQ or academic skills, the GDS was designed to measure physical, emotional, and behavioral development. Later versions of the scales provided more objective observational procedures and acceptable reliability (Bernheimer and Keogh 1988; Knobloch and Pasamanick 1974). In the present study, the GDS was selected for comparability with other studies done in the Chinese population because it has been adopted by the Chinese Pediatric Association, has been validated against a Chinese reference population (Song and Zhu 1987), and is widely used for assessing child development in China and in other countries (Cui et al. 2001; Ke et al. 2004; Zhang and Li 1994; Zhu et al. 2005). Of relevance to the present study, the GDS has been adopted by the WHO for use in resource-poor settings to assess the effectiveness of a program to support health professionals in encouraging parents to stimulate their child’s development in the first 2 years of life (WHO 1999, 2004). This program was introduced to China’s health system in 2001 by WHO and UNICEF. It is considered a high priority for China, particularly for the benefit of those children living in rural areas characterized by difficult access to services and relatively poor economic resources. Recently, the GDS has been used successfully to evaluate developmental gains as a result of the WHO program (Jin et al. 2007), placing importance on the GDS as a complementary tool in health-related research and intervention, as in the present study. Within a Chinese population, there was a significant correlation between developmental assessment at 6–12 months on the GDS and mental development at 6–7 years on the Chinese version of the Wechsler Intelligence Scales for Children (*p* < 0.01) (Zhou et al. 2004).

Children in the cohort who were 2 years of age were administered the version of the GDS for 0- to 3-year-old children, revised by the Beijing Mental Development Cooperative Group (1985) and adapted to the Chinese population. This version of the GDS accurately assesses development of children 4 weeks to 6 years of age (Beijing Mental Development Cooperative Group 1985). The items are grouped into four main categories of functioning: motor behavior, including locomotion, reaching, balance, comprehension, drawing, and hand control; language behavior assessed by means of vocabulary, word comprehension, conversation, and word production; adaptive behavior, including eye–hand coordination, imitation, object recovery, comprehension, discriminative performance, perception, completion, and number conception; and personal and social behavior, including reactions to persons, personal habits, initiative and independence, play responses, and acquired information. Each child is assigned a developmental quotient (DQ) in each of the four specific domains: motor, adaptive, language, and social. The standardized mean (± SD) of the DQ is 100 ± 15. A child with a DQ lower than 85 is considered to have a high probability of some organic impairment (Knobloch and Pasamanick 1974). Scores of 70–84 indicate moderate delay; scores of < 70 indicate severe delay. A score of 84 is the cutoff point for determining normal and developmental delay (Hudon et al. 1998). A study by Jin et al. (2007), which also used the GDS, showed means similar to those in our study.

Testing was conducted by two trained physicians to maximize reliable assessment and valid interpretation. Testers completed a 1-year course at Shanghai Jiaotong University and passed standardized exams to become certified. Therefore, both interexaminer and intraexaminer variability were minimal. Further, the two examiners split the testing by domains, not by subjects. As a result, for any one domain, all subjects were tested by the same examiner.

Research workers abstracted relevant information on covariates from maternal and infant medical records after delivery, such as date of delivery, gestational age, and sex of newborn. Other covariates were derived from questionnaire data on socioeconomic status and environmental exposures.

### Statistical analysis

Age-adjusted DQs in the motor, adaptive, language, and social areas, and the average of these four DQs served as the outcome variables. Initially, in separate linear regression models, associations between each prenatal exposure (PAH–DNA adducts, lead, or mercury in cord blood) and neurodevelopmental DQs were assessed, with adjustment for known potential confounders. The final model included those prenatal exposures (PAH–DNA adducts, lead, or mercury) that were associated with outcomes at the level of *p* < 0.1, along with known potential confounders. Residual plots did not suggest nonlinearities or the need for a transformation of cord adducts. Recent studies have called into question the linearity of the dose–response curve for lead (Lanphear et al. 2005). Moreover, there were influential outliers in the data that nonetheless were within the range seen in other studies. Therefore, we dichotomized cord lead values, as well as mercury values, at the median to minimize the influence of outliers. ETS was assessed as a continuous variable (hours of exposure/day). There were no significant correlations between mercury and adducts (*r* = 0.079, *p* = 0.410); lead and adducts (*r* = −0.052, *p* = 0.586); lead and mercury (*r* = −0.120, *p* = 0.210), or PAH–DNA adducts and ETS (*r* = 0.074, *p* = 0.440) (Spearman’s rho). Thus, collinearity was not an issue.

In separate models, in contrast to adducts and lead, mercury was not associated (*p* < 0.1) with DQ in any domain and was not included in the final multiple regression model: motor area β = −2.258 [95% confidence interval (CI), −6.60 to 2.08], *p* = 0.310; adaptive area, β = −1.202 (95% CI, −6.86 to 4.45), *p* = 0.678; language area, β = −1.254 (95% CI, −6.11 to 3.60), *p* = 0.614; social area, β = −2.366 (95% CI, −6.93 to 2.20), *p* = 0.312; average, β = −1.862 (95% CI, −5.92 to 2.19), *p* = 0.370. Self-reported occupational exposure to chemicals was not associated with DQs. Gestational age, sex, maternal education, and ETS were included in the final model for consistency with other studies. The educational level variable was dichotomized (< high school, ≥ high school). Maternal education level and maternal age were significantly intercorrelated (*r* = 0.217, *p* = 0.008), but maternal education level was the more significant contributor to neurodevelopmental outcomes. We did not adjust for fish intake, a potential source of methyl mercury exposure, because only nine mothers reported consuming any fish during pregnancy. We used logistic regression to estimate the association between prenatal exposures (adducts and lead) and developmental delay, adjusting for the same covariates as in the final multiple regression model.

As noted above, in the present cohort high PAH–DNA adduct levels were associated with decreased birth head circumference (*p* = 0.057) after controlling for potential confounders (Tang et al. 2006). We therefore assessed whether the observed effects of PAH–DNA adducts on DQs were mediated by reduction in head circumference by including birth head circumference in the final regression model.

## Results

The demographic and exposure characteristics of the study subjects are shown in Table 1. The mean PAH–DNA adduct level was 0.32 ± 0.14 adducts/10^8^ nucleotides; 80% of the newborns had detectable adduct levels. Mean lead and mercury concentrations were 3.6 ± 1.59 μg/dL and 7.0 ± 4.43 μg/L, respectively. Table 2 shows the distribution of DQs. All DQ domains were significantly intercorrelated (*p* < 0.01), with *r*-values ranging from 0.56 to 0.86.

The results of multiple regression analysis are shown in Table 3. Increased cord adduct level was inversely associated with decreases in the motor area DQ (β = −16.01; 95% CI, −31.30 to −0.72; *p* = 0.043), language area DQ (β = −16.63; 95% CI, −33.73 to 0.46; *p* = 0.059), and average DQ (β = −14.57; 95% CI, −28.77 to −0.38; *p* = 0.047) after adjusting for cord lead level, ETS, sex, gestational age, and maternal education level. In the same model, high cord blood lead level was significantly associated with decreased social area DQ (β = −6.08; 95% CI, −10.53 to −1.63; *p* = 0.009) and average DQ (β = −4.24; 95% CI, −8.20 to −0.29; *p* = 0.038). There was no indication that the effect of PAH on DQs was mediated by the reduction in head circumference. Birth head circumference did not have a significant effect on the DQs, and its inclusion in the final model did not alter the effect of PAH–DNA adducts on DQs.

The frequency of developmental delay as shown in Table 2 ranged from 9.1% (social) to 13.6% (motor), with 6.4% for the average score. By logistic regression analysis, a 0.1 unit (0.1 adduct per 10^8^ nucleotides) in cord adducts was associated with increased odds of being developmentally delayed in the motor area [odds ratio (OR) = 1.91; 95% CI, 1.22 to 2.97; *p* = 0.004]. An elevated cord lead level was significantly associated with increased probability of being developmentally delayed in the motor area (OR = 3.85; 95% CI, 1.04 to 14.25; *p* = 0.043) and the social area (OR = 7.29; 95% CI, 1.35 to 39.45; *p* = 0.021) (Table 4).

## Discussion

A main finding of the study was that the level of PAH–DNA adducts in newborn cord blood was associated with reductions in 2-year DQs. After adjusting for cord lead level, ETS, and other covariates, increased PAH–DNA adducts were significantly associated with reduced DQs in the motor and language areas and with reduced average DQ. Adducts were associated with increased odds of developmental delay in the motor area. Experimental animal studies have demonstrated that prenatal exposure to BaP, a representative PAH, produces a variety of neurodevelopmental effects, including impairment of long-term potentiation, a cellular correlate of learning and memory, in offspring (Wormley et al. 2004a). In epidemiologic studies, prenatal exposure to PAHs has been shown to be associated with adverse birth outcomes (Choi et al. 2006; Perera et al. 1998, 2006) and with a decrement in the mental development index on the Bayley Scales of Development at 3 years of age (Perera et al. 2006). As noted above, several prior studies have shown that high PAH–DNA adduct levels were associated with reduced fetal growth (Perera et al. 1998; Tang et al. 2006), including decreased birth head circumference (*p* = 0.057) in the present Chinese cohort, after controlling for potential confounders (Tang et al. 2006).

Because PAH–DNA adduct levels reflect individual variation in exposure, absorption, metabolic activation, and DNA repair, the level of PAH–DNA adducts provides a biologically relevant dosimeter of exposure. Although we can hypothesize that adduct formation resulting in mutations or apoptosis during critical windows of brain development might adversely affect child development, here we are considering adducts as a biologically relevant measure of exposure. We note that the cord adduct levels in the Tongliang study were significantly higher than in New York City or Polish newborns, consistent with the higher PAH exposure in Tongliang (Perera et al. 2005). Specifically, the mean adduct concentration was 45% higher in the Tongliang newborns compared with New York City newborns (Perera et al. 2005). As in several prior studies (Perera et al. 2005), adducts in cord blood were not correlated with ETS exposure.

In the present study, lead was significantly associated with reductions in social and average DQs. Lead was also associated with increased odds of developmental delay in the motor and social areas. These findings are consistent with recent studies showing low-level effects of lead: cord lead levels in the range of 0.82–12.93 μg/dL (mean, 3.6 μg/dL) found in this study were significantly associated with decreased social area DQ and decreased average DQ. As noted above, low-level lead exposure as measured by cord blood lead is associated not only with lower IQ (ATSDR 2005; Canfield et al. 2003; Lanphear et al. 2005; Surkan et al. 2007) but also with antisocial and delinquent behavior (Needleman et al. 1996).

In this study, prenatal mercury exposure had no observable effect on the GDS scores. This nonpositive finding may be attributable to the relatively low levels of mercury in cord blood in the present cohort (mean ± SD, 7.0 ± 4.42 μg/L) compared with the level of methylmercury in the Faroe Island cohort (mean, 22.9 μg/L) that reported adverse effects of prenatal mercury exposure (Grandjean et al. 1997). In the present cohort, only nine mothers consumed any fish or shellfish, which is the most important source of mercury.

A limitation of the study is that we do not have data on postnatal blood PAH–DNA, lead, and mercury levels to allow us to examine the impact of postnatal exposure on 2-year cognitive development. Because the power plant was not shut down until May 2004, the subjects continued to receive seasonal exposure to the plant emissions after birth. However, fetal development is considered to be the period of greatest susceptibility to PAHs and lead. Another limitation of the present study is the small sample size, precluding evaluation of interactions between pollutants. Moreover, all babies were exposed to PAHs from the power plant in both the second and third trimesters, and we were therefore unable to separate exposures by trimester. The deficits in development at 2 years of age may be educationally meaningful because compromised function at an early age may have a negative impact on subsequent school performance (Drillien et al. 1988). As noted above, within a Chinese population, there was a significant correlation between developmental assessment at 6–12 months on the GDS and mental development at 6–7 years on the Chinese version of the Wechsler Intelligence Scales for Children (*p* < 0.01) (Zhou et al. 2004). Continued follow-up of the present cohort will determine whether prenatal PAH exposure is a risk factor for subsequent cognitive effects.

## Conclusion

The present results indicate that among children living in Tongliang, Chongqing, *in utero* exposure to PAHs from a coal-fired power plant adversely affected motor and language development. Prenatal exposure to lead adversely affected social development. Because coal-fired power plants currently produce 75% of China’s electricity and most new plants in China are being built to burn coal, the results from the Tongliang study are relevant to the development of other children living in China and have significant implications for policies concerning energy and public health.

## Figures and Tables

**Table 1:** Demographic characteristics of the study sample (*n* = 110).^a^

Demographic characteristic	Mean ± SD (range)
Maternal age (years)	25.2 ± 3.2 (20.34–34.28)
Maternal education (%)	
< High school	43.6
≥ High school	56.4
Cord adducts (adducts/10^8^ nucleotides)	0.32 ± 0.14 (0.125–0.812)
Cord lead (μg/dL)	3.60 ± 1.59 (0.82–12.93)
Cord mercury (ppb)	7.0 ± 4.43 (2.28–39.72)
Prenatal ETS exposure (hr/day)	0.293 ± 0.586 (0–5)
Sex of newborn (% female)	50.9
Gestational age (days)	277.3 ± 11.3 (224–294)

a This subset did not differ significantly with respect to demographic characteristics from the group not included in the analysis [e.g., maternal age (*p* = 0.579), gestational age (*p* = 0.232), birth weight (*p* = 0.614), birth length (*p* = 0.564), or birth head circumference (*p* = 0.563), by *t*-test]. However, maternal education differed between the groups (*p* = 0.056).

**Table 2:** Distribution of GDS DQ scores (*n* = 110).^a^

	Mean ± SD (range)	Normal [*n* (%)]	Developmental delay [*n* (%)]
Motor area	97.53 ± 11.47 (65–135)	95 (86.4)	15 (13.6)
Adaptive area	98.71 ± 14.90 (50–124)	96 (87.3)	14 (12.7)
Language area	102.10 ± 12.83 (56–122)	99 (90.0)	11 (10.0)
Social area	99.40 ± 11.79 (57–121)	100 (90.9)	10 (9.1)
Average	99.42 ± 10.74 (57–120)	103 (93.6)	7 (6.4)

a Normal, > 84; developmental delay, ≤ 84.

**Table 3:** Results of multiple regression analyses^a^ of GDS DQ scores at 2 years of age and PAH–DNA adducts and cord lead in newborn cord blood (*n* = 110).

	Motor		Adaptive		Language		Social		Average	
	β (95% CI)	*p*-Value	β (95% CI)	*p*-Value	β (95% CI)	*p*-Value	β (95% CI)	*p*-Value	β (95% CI)	*p*-Value
Cord adducts (adducts/10^8^ nucleotides)	−16.01 (−31.30 to −0.72)	0.043	−15.51 (−35.63 to 4.61)	0.134	−16.64 (−33.73 to 0.46)	0.059	−9.29 (−25.28 to 6.70)	0.258	−14.58 (−28.77 to −0.37)	0.047
Cord lead (H/L)^b^	−3.72 (−7.98 to 0.53)	0.089	−3.59 (−9.20 to 2.01)	0.212	−4.34 (−9.10 to 0.43)	0.077	−6.08 (−10.53 to −1.63)	0.009	−4.24 (−8.20 to −0.29)	0.038

a Model included sex, gestational age, maternal education, and ETS as covariates.

b Using median of cord lead (3.5 μg/dL) as cutoff for H/L (high/low).

**Table 4:** Results of logistic regression analyses of GDS scores at age 2 and adducts and cord lead in newborn cord blood (*n* = 110).^a^,^b^

	Motor		Adaptive		Language		Social		Average	
	OR (95% CI)	*p*-Value	OR (95% CI)	*p*-Value	OR (95% CI)	*p*-Value	OR (95% CI)	*p*-Value	OR (95% CI)	*p*-Value
Cord adducts (adducts/10^8^ nucleotides)	1.91 (1.22 to 2.97)	0.004	1.16 (0.76 to 1.76)	0.500	1.31 (0.84 to 2.05)	0.234	1.52 (0.93 to 2.50)	0.095	1.67 (0.93 to 3.00)	0.088
Cord lead (H/L)^c^	3.85 (1.04 to 14.25)	0.043	1.43 (0.43 to 4.68)	0.559	2.70 (0.70 to 10.50)	0.150	7.29 (1.35 to 39.45)	0.021	4.25 (0.70 to 25.89)	0.116

a Model included sex, gestational age, maternal education, and ETS as covariates.

b The odds ratios for cord adducts represent the effect of a 0.1-unit increment in cord adducts.

c Using median of cord lead (3.5 μg/dL) as cutoff [H/L (high/low)].
